# Is the Role of Physicians Really Evolving Due to Non-physician Clinicians Predominance in Staff Makeup in Sub-Saharan African Health Systems? Comment on "Non-physician Clinicians in Sub-Saharan Africa and the Evolving Role of Physicians"

**DOI:** 10.15171/ijhpm.2016.80

**Published:** 2016-07-02

**Authors:** Mohsin M. Sidat

**Affiliations:** Faculty of Medicine, University Eduardo Mondlane, Maputo, Mozambique.

**Keywords:** Physicians, Non-physician Clinicians (NPCs), Health Workforce, Human Resources for Health, Sub-Saharan Africa, Physician Role, Physician Training, Physician Competencies

## Abstract

Health workforce shortages in Sub-Saharan Africa are widely recognized, particularly of physicians, leading the training and deployment of Non-physician clinicians (NPCs). The paper by Eyal et al provides interesting and legitimate viewpoints on evolving role of physicians in context of decisive increase of NPCss in Sub-Saharan Africa. Certainly, in short or mid-term, NPCs will continue to be a proxy solution and a valuable alternative to overcome physicians’ shortages in sub-Saharan Africa. Indeed, NPCs have an important role at primary healthcare (PHC) level. Physicians at PHC level can certainly have all different roles that were suggested by Eyal et al, including those not directly related to healthcare provision. However, at secondary and higher levels of healthcare, physicians would assume other roles that are mainly related to patient clinical care. Thus, attempting to generalize the role of physicians without taking into account the context where they will work would be not entirely appropriate. It is true that often physicians start the professional carriers at PHC level and progress to other levels of healthcare particularly after clinical post-graduation training. Nevertheless, the training programs offered by medical institutions in sub-Saharan Africa need to be periodically reviewed and take into account professional and occupational roles physicians would take in context of evolving health systems in sub-Saharan Africa.

Health workforce shortages in sub-Saharan Africa are widely recognized, particularly of physicians, which has been implicated for relatively high rates of morbidity and mortality in the continent.^1^ The urgency to overcome this situation showed the way for the development of training programmes and deployment of other categories of health workers.^2^ These other categories were trained to provide tasks that were usually performed by physicians and nurses, and the process of delegation of tasks to these cadres is known as *task-shifting.*^1,2^ The shift or delegation of tasks to these other cadres was mainly aimed to rapidly increase access, coverage and scope of health services within mainly poorly served rural areas of sub-Saharan Africa.^3^ Diverse designations have been used for these cadres in different countries, depending on tasks they were trained to perform and the role they were assigned when deployed within the level of healthcare services.^4^ These cadres are often categorized as substitute health workers (SHWs),^3^ auxiliaries, clinical officers, health officers, physician assistants, nurse practitioners, nurse clinicians, associate clinicians, Non-physician clinicians (NPCs)^2^ or mid-level providers (MLPs).^4-9^ These diverse designations are often used interchangeably to mainly refer to health cadres who are submitted to shorter and faster training programmes to perform well-defined set of tasks that commonly are of responsibility of physicians and/or nurses.^2,7^ However, there is no formal or widely agreed single designation for NPCs that goes with the International Standard Classification of Occupations.^6^ Additionally, there are still unresolved issues affecting NPCs in many countries such as of accreditation, professional recognition and carrier progression.^3,6^ These persisting issues, for example, limit mobility of NPCs across the countries and reduce opportunities of employability mainly to public health sector. Regardless of all these issues, NPCs play an important role in health services provision in context of shortages of physicians and other types of health workers in many parts of the world, particularly in sub-Saharan Africa.^2,3,6,7^ Overall, NPCs are trained in lesser time, cheaper to employ and tend to stay in underserved areas (rural and remote areas) for longer periods.^8^

The paper by Eyal et al^9^ provides interesting viewpoints on evolving role of physicians in context of decisive increase of NPCss in sub-Saharan Africa. The view that the “*development in NPC deployment should unfold in parallel with strategic rethinking about the role of physicians and with critical innovations in physicians education and in-service training*” is, in my view, legitimate. Certainly, in short or mid-term, NPCs will continue to be a proxy solution and a valuable alternative to overcome physicians’ shortages in sub-Saharan Africa. Indeed, as referred by Eyal et al,^9^ “*NPCs deployment has finally gained full acceptance by local health sector leaders.*” Their paper presents compelling evidence of increasing predominance and diversity of roles of NPCs in rural and underserved settings in sub-Saharan Africa, but also lists several challenges that persist within health systems “*to maximize the effectiveness of NPCs.*”^9^ Some challenges presented by Eyal et al,^9^ such as those related to curricula, teaching facilities and standardized decision-making algorithms, can be successfully addressed with decisive policies and approaches by health sector leadership from sub-Saharan countries. In fact, depending on contextual factors and needs of health services, the curricula and teachings strategies can be developed and implemented. Additionally, appropriate clinical algorithms specific for diversity of NPCs implementers can be produced based upon national and even international experiences and expertise. However, there are challenges that certainly are more problematic and relatively more complex to resolve, such as: career development prospects; appropriate legislation and mechanisms for professional recognition; and strategies for resolution of persisting conflicts between NPCs and other health workers. In fact, in my view, significant policy gaps still exists in sub-Saharan African countries, for instance regarding legislation and policies related to professional progression pathways and scopes of work of NPCs.^6^ These persisting gaps contributes for workplace frustrations and relational conflicts in place of work with negative impact in services provision.^6^ Thus, these persisting challenges need urgent attention in view of short-midterm dependency on NPCs by most sub-Saharan African countries.^5,6,8^ There is limited capacity to train physicians in sub-Saharan African countries in view to attend the short-midterm needs of health system in these countries.^1,5^

Eyal et al recognize that “*even in a predominantly NPC-based health system, physicians remain highly necessary*” and, therefore, the physicians role within the health system in sub-Saharan African countries need to be reviewed and updated.^9^ To redefine the role of physicians in context of NPCs predominance in health system, Eyal et al^9^ recur to World Health Organization (WHO) health system building blocks as a possible framework to delineate set of competencies. However, the set of competencies and the role physicians that were suggested fail in taking into account contextual factors of the level of health system physicians will be working. The level of health system where physicians are deployed at start of their professional carriers would determine the occupational profile and set of skills and competencies they need to have when graduating from Medical Schools. For example, after graduation, if the physicians have to serve as clinicians at primary healthcare (PHC) level, then the sets of skills and competencies they will need would have to be aligned to set of clinical, administrative and/or other services they are expected to provide at this particular level. The set of skills and competencies will clearly differ from the ones a physician will need when deployed at secondary or higher levels of healthcare services. In my view, the sets of skills and competencies suggested by Eyal et al^8^ are somehow appropriate for physicians graduating from medical schools and deployed at health facilities of PHC level where NPCs are often deployed and in relatively greater number in most sub-Saharan African countries.^6,7^ Thus, I agree that Institutions training physicians that will be deployed at PHC level should revisit the professional and occupational profile of their degrees and incorporate skills and competencies that are aligned to context of working where NPCs are significant part of health teams. Medical training Institutions in each sub-Saharan country need to be aware of possible roles their graduates can have with their health system and make appropriate adjustments of set of skills and competencies. The framework suggested by Eyal et al^9^ can be one of the tools that can help leadership of medical training institutions to envision possible roles their trained physicians can play at PHC level and/or other levels of health system in sub-Saharan Africa. Nevertheless, all efforts to redefine the role and sets of skills and competencies of physicians should also take into account other available guidelines and frameworks.^10-13^

As referred before, in short-midterm, the health systems of sub-Saharan African countries will be dominated by NPCs mainly at PHC level sub-system. The NPCs provide best option for sub-Saharan African countries and others.^3^ Indeed, NPCs are a kind of a quick-fix for these countries as they require shorter training times, lesser resources for their training and represent certainly a less-expensive option for basic health services provision for already financially burden health systems.^2,3,6,7^ However, the health system leadership in sub-Saharan Africa need to resolve swiftly pending issues related to NPCs such as those related to legislation, professional progression pathways and clear scopes of work.^6^

Finally, it does not seem necessary for NPCs to be trained alongside with physicians at same training institutions as suggested by Eyal et al,^9^ but medical training institutions should perhaps be aware of set of skills and competencies of different NPCs that exists in their health system and incorporate this knowledge when developing physicians training *curricula*. Many medical training institutions offer only physician training courses and seems quite challenging for many conservative Institutions to also include training of NPCs. In many sub-Saharan countries, NPCs training occurs outside medical training institutions. However, all efforts should be made to take advantage of the opportunities given by clinical training in health facilities where physicians-trainees, NPCs-trainees and other health cadres in training co-exists. It is of utmost importance to create an environment of training at this level that supports team-building and inter-professional relationship skills and competencies learning. Both, physicians and NPCs, have both types of cadres have roles to play which sometimes overlap and requires mutual understanding and effort for harmonious and well-productive working environment. Thus, to conclude it seems appropriate to quote Fulton et al^14^ who said: “*by providing healthcare services at the productively efficient skill mix–the mix that produces the maximum number of healthcare services at a given quality and cost–more healthcare services are going to be accessible and affordable to populations seeking care. Task-shifting is a policy option that should be considered to help achieve productive efficiency and provide access to services that otherwise might not be available. A more productively efficient skill mix will partially dampen the effect of health workforce needs-based shortages and better enable countries to meet the health-related United Nations Millennium Development Goals.*”

## Ethical issues

Not applicable.

## Competing interests

Author declares that he has no competing interests.

## Author’s contribution

MMS is the single author of the paper.
